# Black Lives Matter: A Decomposition of Racial Inequalities in Oral Cancer Screening

**DOI:** 10.3390/cancers13040848

**Published:** 2021-02-17

**Authors:** Benjamin Lam, Lisa M. Jamieson, Murthy Mittinty

**Affiliations:** 1 School of Public Health, The University of Adelaide, Adelaide 5005, Australia; lisa.jamieson@adelaide.edu.au (L.M.J.); murthy.mittinty@adelaide.edu.au (M.M.); 2 Australian Research Centre for Population Oral Health, The University of Adelaide, Adelaide 5005, Australia

**Keywords:** interaction analysis, Oaxaca decomposition, decomposition, IPTW, causal analysis, marginal structural models, MSM

## Abstract

**Simple Summary:**

Black Lives Matter has highlighted the increased social discrepancies that exist not only in the context of social justice but also in public health. Oral cancer screening is not exempt from disparity, with Black Americans less likely to seek screening leading to higher incidence and worse outcomes of oropharyngeal cancers. We investigate interaction analysis and Blinder–Oaxaca decomposition as tools to guide policy to address this disparity. Using National Health and Nutrition Examination Survey (NHANES) data from 2011–2018 we find that being both in poverty and Black results in sub-additive interaction, which is further deconstructed into differences in higher education levels and poverty status.

**Abstract:**

(1) Background: The Black Lives Matter movement has highlighted the discrepancies in public health in regard to race. This study aims to investigate tools that can be used to analyze and investigate this discrepancy, which can be applied to policymaking. (2) Methods: National Health and Nutrition Examination Survey (NHANES) data from 2011–2018 was combined (*N* = 22,617) to investigate discrepancies of oral cancer screening in Black Americans. We give examples of counterfactual techniques that can be used to guide policy. Inverse probability treatment weighting (IPTW) was used to remove all measured confounding in an interaction analysis to assess the combined effect of socioeconomic status and race. Blinder–Oaxaca decomposition was then used to investigate the intervenable factors associated with differences in race. (3) Results: Sub-additive interaction was found on additive and multiplicative scales when all measured confounding was removed via IPTW (relative excess risk due to interaction (RERI)(OR) = −0.55 (−0.67–−0.42)). Decomposition analysis found that 32% of the discrepancy could be explained by characteristics of higher education and poverty status. (4) Conclusions: Black Americans in poverty are less likely to seek oral cancer screening than the additive likelihood would suggest. Blinder–Oaxaca decomposition is a strong tool to use for guiding policy as it quantifies clear breakdowns of what intervenable factors there are that would improve the discrepancy the most.

## 1. Introduction

The Black Lives Matter (BLM) movement has recently reinvigorated discussions of disparity between African Americans and their White counterparts. Whilst the movement finds its roots in the long history of civil rights movements dating from the origins of the United States, this renewed focus on police brutality and social injustice has increased awareness of the differences that exist in health practices across the country and the reasons for this inequity. Yet this is not a new problem, many have written about racism being a matter of public health and that epidemiologists and health professionals have an ethical responsibility to address this [1]. Thus this study’s contribution to the movement is to investigate differences and discrepancies in oral cancer screening amongst Black Americans.

Disparities persist in oral health practices, in particular oral cancer screening among Black Americans [2,3]. Despite advances in treatments and increased awareness increasing the overall prospects of those diagnosed with oropharyngeal cancer (OPC), Black Americans, in particular, but not limited to males, still have increased incidence of diagnoses and lower survival rates than their non-Black equivalents [4]. It is clear from prior studies that closing the gap in oral cancer screenings between Black and non-Black Americans is of great importance in order for Black Americans to achieve commensurate survival outcomes as non-Black Americans with OPC [5,6], but without insights into what actual factors can be modified via policy, it becomes a difficult problem to solve. We believe that race and racial difference itself is both a social construct and a culmination of a multitude of factors that may be intervenable.

Socioeconomic status (SES) is also often linked to lowered health outcomes globally and within the United States. Social disadvantage is a constant theme in oral cancer research [3,6,7,8,9] and cancer research in general [10] with empirical evidence demonstrating lower oral cancer screening (23% for Black Americans compared to 50% for Whites), worse outcomes of cancer diagnosis (OR of Grade 2 tumor 1.9 (1.5–2.3) for Blacks compared to Whites) [6] and cancer survival (five-year survival % for all stage at diagnosis 39.5% for Blacks and 61.8% for Whites) [4]. The overarching question is if identifying as Black increases the risk of not having an oral cancer screening, is this increase magnified if the individual is both Black and in a low SES group.

Interaction analysis is often used to investigate the excess risk of having two contributing variables on the outcome. In other words, how less likely is it that an individual will seek out an oral cancer screening if they are both Black and in poverty as opposed to just one of those categories. By using sufficient cause analysis techniques to create counterfactual contrasts in a marginal structural model (MSM), interaction analyses for excess risk can be calculated while accounting for all measured confounding. Inverse Probability Treatment Weighting (IPTW) is commonly used to create the counterfactual weights used to create MSM’s. By using IPTW it is possible to nullify the measured confounders to create a situation where the model is isolated on the main outcome and explanatory factors only. It also has a significant advantage in that it is very simple to run as a single extra step to a linear model.

Interaction analysis is useful when one is interested in estimating the risk differentials of poverty on cancer screening within the levels of race, however, this does not inform policymakers as to what modifiable factors would realistically reduce the risk. In order to look at the main confounders that contribute to the gap in differentials within the counterfactual framework, we consider the use of Blinder–Oaxaca decomposition.

Blinder–Oaxaca decomposition is a technique primarily used in econometrics and social science to investigate differences between groups, usually race or sex but not limited to those. With the development of Oaxaca decomposition extending from its origins in linear regression to use with a variety of different models, its use in epidemiology and health research has become increasingly common. Its ease of use and implementation in most major statistical software packages allows it to be a very strong tool if used and interpreted correctly.

Thus, this study aims to use a multifaceted approach by first investigating the non-intervenable factors of low SES status and race to investigate the relative excess risk due to interaction (RERI) by first applying marginal structural model interaction analysis. Non-intervenable factors will then be broken into intervenable factors that can be used to guide policy by deconstructing race to find the intervenable factors that can help guide policy and close the gap. As such we intend to examine intervenable systemic factors that lead to this difference and the policy changes that could mitigate such challenging issues.

## 2. Results

The results will be divided into three sections: (1) basic descriptive statistics and unadjusted logistic regression and RERI; (2) inverse weighted RERI for non-intervenable factors and: (3): Oaxaca decomposition for intervenable factor analysis. In all analyses, P-values were for reference only and were not used for inference. All results are given to two significant figures.

### 2.1. Descriptive Statistics and Standard Multivariate Logistic Regression

Table 1 shows descriptive statistics of factors comparing Black to Non-Black individuals. The poverty income ratio (PIR) varies significantly between the groups, where Blacks are closer to the poverty line indicated by a PIR value of 1. Overall household income is similar for the mid group of $25k–$75k annually, however, the major discrepancies are in the low and high-income groups where the Black population has increased percentage in the former compared to the latter. Overall wealth disparity is skewed in opposite directions between the two groups. Similar results are found in the education level indicator. Within the health factors, there is a significant difference across all factors with Black Americans generally performing worse than their non-Black counterparts. Self-reporting of having an oral cancer check explained did not differ meaningfully between Blacks and non-Blacks.

In Table 2 the logistic regression with the outcome of having an oral cancer check shows a significant drop in odds from non-Black to Black with an odds ratio of 0.40. Factors of having the benefits of a cancer check being explained, as well as education level, household income, and being insured resulted in significantly higher odds of having an oral cancer check. Of the chosen variables, only current smoker status had 95% confidence intervals overlapping an odds ratio of 1 (0.71–1.18).

Survey weighted interaction analysis with no further adjustments in Table 3 shows a slight super-additive interaction on both the additive (RERI > 0) and multiplicative scale. However in this confounded model, even adjusting for survey weighting the confidence intervals of the multiplicative scale overlap the critical ratio value of 1, casting doubt on the true direction of excess risk.

### 2.2. Inverse Probability Treatment Weighted (IPTW) Interaction Analysis

IPTW weights are created on the probability of being in poverty and used in conjunction with the 8-year Mobile Examination Center survey weights.

The survey weighted un-confounded model suggests sub-additive RERI on both risk ratio and odds ratio, as well as negative multiplicative interaction. The inverse probability treatment weighting creates the counterfactuals and is used as a method to remove measured confounding from the analysis which has greatly improved the variability of the point estimate of relative excess risk, this can be seen in the narrowing of the confidence intervals. As the dataset does not allow an interpretation of causal inference, as well as poverty and Black being non-intervenable factors, it should be interpreted as effect modification of environmental factors. There is reasonably strong evidence that removing observed confounding, being both Black and in poverty, increases the relative risk of not having an oral cancer check compared to when being in just one of these groups.

### 2.3. Non-Linear Blinder–Oaxaca Decomposition

Table 4 shows the decomposition of the gap between Blacks and non-Blacks with the outcome of having received an oral cancer check. Unlike the effect measure modification, in this analysis, a clear breakdown of which intervenable factors are causing the difference between the two groups can be observed. The percent contribution is calculated from the decomposed estimate divided by the raw difference. From the model, the explained differences due to characteristics (being Black vs. non-Black) make up 31.79% of the difference in oral cancer checks whilst the differences due to coefficients or the unexplained component makes up the remaining 68.21%.

Table 5 shows the complete Blinder-Oaxaca decomposition. The difference due to coefficients equates to the difference explained if non-Black coefficients were applied to Black characteristics. Of the difference due to coefficients, a significant portion was explained by SES factors including higher levels (33%, 38%) of income as well as insurance status (51%). Unmeasured factors (the constant term) made up −79% of this difference, having a significantly large negative proportion shows that with the counterfactual of the Black group having the same coefficients as the non-Black group, with all coefficients set to zero the gap would decrease by 79%.

**Figure 1:**
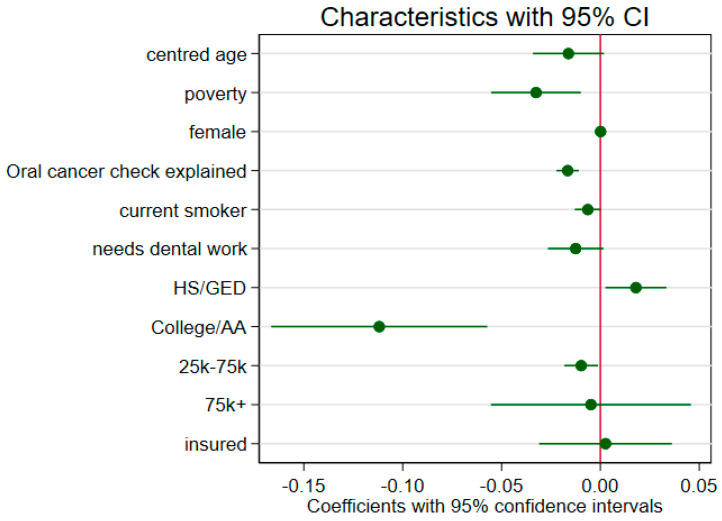
Coefficient plot of the decomposed characteristics with 95% confidence intervals. HS/GED—High School/General Educational Development

The difference due to characteristics equates to the difference explained if Blacks had the same characteristics as non-Blacks. Of the proportion explained due to characteristics, the largest indicators of difference came from having a college education and poverty status. This can be interpreted as if Blacks had the same levels of education and poverty status as non-Blacks, the overall racial gap in oral cancer checks would decrease by 41% and 12% respectively which can be seen clearly in Figure 1.

## 3. Discussion

Previous studies have demonstrated that Black Americans are not being screened for oral cancers and as a result are subject to an increased risk of developing these cancers compared to non-Black Americans [2,3,5,11]. This study is not intended as a confirmation of these facts, but rather an exploratory analysis into techniques that can be used to inform policy and make a systematic change to bridge these inequities. As such, by applying a multifaceted design this study first confirmed the existence of this difference and built upon it by including SES level in an interaction analysis of marginal structural models, and then examined in-depth factors contributing to this difference by using Blinder–Oaxaca decomposition. As far as we are aware, this is the first study that has applied Oaxaca decomposition to oral cancer screening, though its use has been found in studies of disparities in other cancer screenings such as lung, breast, and prostate [12,13,14].

### 3.1. Race and Socioeconomic Status

The initial interaction analysis demonstrated a sub-additive interaction on the additive scale and a negative interaction on the multiplicative scale. The consequences of such can be interpreted as an increase in excess risk when an individual is a member of both groups, Black and low SES level (poverty) as opposed to just one. Prior literature on SES and race tended to focus on SES simply as an extra explanatory factor [6,7], whereas the additional step in our analysis enabled the combination of both to be tested. The complexity of SES and race is that they are both heavily intertwined yet still theoretical proxies of distinct exposures. Evidence of this is apparent when considering how racial differences in health outcomes appear across all SES strata [15]. Our analysis shows that SES is not simply an explanatory factor, but rather complementary to the problem at large. The methodology of using marginal structural models by taking inverse probability weights to remove all measured confounding greatly stabilizes the variance when calculating measures of interaction when compared to the confounded models. 

### 3.2. Blinder–Oaxaca Decomposition to Guide Policy

The application of Oaxaca decomposition is ideal for studies comparing racial contrasts. Race is not intervenable. The complexities of race and health research have been deeply explored by various authors and all have come to a similar conclusion, racial differences are a problem of public health policy [1,16,17]. Some argue that it is systematic [18]; Blacks are just not receiving the same level of care, others argue that it is a problem of community [19]; there are inadequacies in the communities’ approach or understanding of health or some combination of both. It is here that Oaxaca decomposition shows its true worth. By breaking down the components into those explained by differences and those unexplained by differences, the exact problems that need to be intervened upon in order to make a change in health outcomes becomes apparent. In this study, there was strong evidence that the difference in higher education level amongst Blacks and non-Blacks was the largest contributor to the overall difference in oral cancer screening. This contribution was calculated at 41%, indicating that if Blacks had the same education level as non-Blacks, the explained gap would close by 41%. This is a significant amount, especially if the relative difference compared to other factors is taken into account. As Oaxaca decomposition is able to give a direct quantifiable difference that can be intervened on, its utility as a tool that policymakers should increasingly use to guide decision making on intervenable factors becomes apparent. 

### 3.3. Limitations, Recommendations, and Extensions

As with any statistical method, there are limitations. Causal inference cannot be made as the research design is cross-sectional, which does not allow for the necessary time-causation direction. Measurement error or misspecification in the survey questionnaire were not considered, which could increase bias. As the survey was not created with our research question and methodology in mind, it did not include some factors that theoretically are important. For instance, questions on community and belonging, or racism indicators. There was a significant non-response for oral cancer screening explained with only 54% of the full cohort responding. All other variables had high response rates (>90%).

The use of MSM in the interaction analysis assumes that all confounding is accounted for and that there is no unmeasured confounding, a situation which is highly unlikely. Techniques in sensitivity analysis in unmeasured confounding for interaction analysis exist but are seldom used due to relative complexity compared to more developed techniques such as Vanderweele and Ding’s E-value for risk ratios [20,21,22]. 

At its core, Oaxaca decomposition works best with well-defined and strong contrasting groups. There are limited publications on decompositions of differences between edge groups as strong counterfactuals cannot be created in such cases. There are also issues with the choice of reference group altering directional interpretation, otherwise known as the “index number problem” [23]. It is recommended that analysis is also conducted in reverse, a function most software implementations already possess. Finally, the interpretation of Oaxaca decomposition should be carefully assessed. Some studies only present the differences due to characteristics and do not attempt to assess the unexplained components or constant terms [24,25]. With further development of Oaxaca decomposition moving towards including time-varying covariates, this could be an interesting tool to investigate group differences over time [26].

Recent studies suggest a large incidence of Human Papillomavirus (HPV) within the American population as well as a link between HPV and OPC [7,27,28]. Oral cancer screening does not prevent OPC, but rather it is a means to early detection which increases the chance of survival [11,29]. Given that HPV vaccination is a relatively simple procedure, an investigation of non-intervenable and intervenable factors on racial discrepancies in HPV vaccination would be a worthwhile extension to this current analysis.

## 4. Materials and Methods 

### 4.1. Data Source, Outcome Variable, Exposures, and Confounders

Data was sourced from the National Health and Nutrition Examination Survey (NHANES) collected by the Center for Disease Control and Prevention (CDC). NHANES is an expansive set of cross-sectional studies that assesses a large array of health factors of adults and children in the United States. Publicly available datasets were analyzed in this study. This data can be found here: (https://wwwn.cdc.gov/nchs/nhanes/Default.aspx (accessed on 24 February 2021)). Topics include general factors, self-assessed health factors, as well as clinical and lab testing results [30]. Data comes in waves every two years; this study merged the demographic, oral health, smoking-cigarette use, and health insurance of cycles from 2011–2018. We assessed observations at ages 18–80+, with a total sample of 23,817 respondents, 22,617 remaining after removing subjects with significant missing responses. Survey weighting from each 2-year cycle was combined to create an 8-year survey weight. Mobile Exam Center (MEC) weights were used due to the inclusion information collected in the MEC exam under the recommendation of NHANES [31]. The University of Adelaide Human Research Ethics Committee waives the requirement of ethical approval for de-identified secondary data analysis from publicly available repositories.

The outcome of oral cancer screening was chosen as it exhibits a strong difference between Blacks and non-Blacks but also within socioeconomic measures. Within NHANES it is defined as “oral cancer screening where the doctor pulls the tongue” which is likely to help elicit memories of the screening being undertaken.

The main exposures of socioeconomic status and race were chosen as they are not intervenable allowing for closer analysis via decomposition techniques. The poverty income ratio (PIR) is calculated by dividing the household’s annual income by the poverty line. Any value below 1 indicates the household earns less than the poverty line and values above 1 indicate living above the poverty line. PIR was chosen as a proxy for socioeconomic status where a PIR < 1 would be considered in poverty and of low-socioeconomic status however alternative cut-lines could be evaluated. Furthermore, arguments of the intervenability of socioeconomic status were rejected due to lowered opportunities in social mobility, especially amongst Black Americans [32]. Race was dichotomized into non-Hispanic Blacks and non-Blacks encompassing all other ethnicities. This could also be evaluated to not include ethnic minorities which may show similar disadvantage to Blacks. 

Intervenable factors were considered as confounders. General confounders included gender (male, female), and age (18–80 years). Socioeconomic confounders included poverty income ratio (1–5), annual household income (<25$k, 25$k–75$k, 75$k+), education level (less than high school, high school/GED, College/AA degree), marital status (married/defacto, divorced/separated, single), insurance status (yes, no), and children in the household (yes, no). Health-related confounders included current smoking status (yes, no), requiring a dental procedure but not being able to access it (yes, no), and benefits of oral cancer screening explained (yes, no). Of these, marital status and children in the household were found to not contribute any meaningful difference and were removed from the multivariate analysis. All covariates had a response rate of above 90% apart from the benefits of oral cancer screening explained which had a response rate of 54%. As this study’s primary purpose was an exploration of methods, no missing data imputation was conducted, and complete case analysis was used, however, even with consideration to non-responses we believe that the dataset is more than adequately sized for this methodology. 

### 4.2. Statistical Methods

All statistical analysis was conducted using Stata/SE 15.0 (StataCorp).

#### 4.2.1. Standard Logistic Regression and Interaction Analysis

Descriptive statistics and bivariate analysis comparing racial groups were conducted using chi-square tests for categorical variables and *t*-tests for continuous variables. A logistic regression with the outcome of receiving an oral cancer screening was conducted with all intervenable and non-intervenable variables. This was conducted to gain an understanding of which factors could be used to predict oral cancer screening. It also served as an exploration for the confounded interaction analysis as both Black and poverty status indicated decreased odds in receiving an oral cancer screening. 

Relative excess risk due to interaction (RERI) and multiplicative interaction analysis is an approach that assesses the direction of increased (or decreased) risk due to an interaction. In this case, two dichotomous variables were used to assess whether being both Black and in poverty decreases the likelihood of receiving an oral cancer screen. Interaction analysis is simple to run and easy to interpret. The reasoning is described elsewhere [33,34,35] but briefly, a log-linear model with dichotomous predictors A and B with interaction term AB.

$$\log[P(Y=1|A=a,\;B=b)]=\beta_{0}+\beta_{1}a+\beta_{2}b+\beta_{3}ab$$

where $\exp\left(\beta_{v}\right)$ is the risk ratio of each covariate.

Interaction on the multiplicative scale (IMS) can be assessed by the ratio of the risk ratio of the interaction against the product of the two independent risk ratios:

$$IMS=\frac{RR_{A_{1}B_{1}}}{RR_{A_{1}}RR_{B_{1}}}$$

Due to the interaction of the model being the same as the ratio of the risk ratios of the independent covariates [34], the exponent of $\beta_{3}$ can be used as a quick indicator of multiplicative interaction.

$$\begin{array}{l} \exp\left(\beta_{3}\right)\\ =\frac{P\left(Y=1|A=1,B=1\right)/P\left(Y=1|A=0,B=0\right)}{P\left(Y=1|A=0,B=1\right)/P\left(Y=1|A=0,B=0\right)P\left(Y=1|A=1,B=0\right)/P\left(Y=1|A=0,B=0\right)} \end{array}$$

$$\frac{P\left(Y=1|A=1,B=1\right)*P(Y=1|A=0,B=0)}{P\left(Y=1|A=1,B=0\right)*P(Y=1|A=0,B=1)}$$

$$=\frac{RR_{A_{1}B_{1}}}{RR_{A_{1}}RR_{B_{1}}}$$

The IMS indicates multiplicative interaction when IMS>1 and negative when IMS<1 as it is a ratio. When IMS=1 the interaction term does not perform any better than the two singular terms. 

As odds ratios will approximate risk ratios with an outcome that is rare, regressions that give odds ratios can also be used; a popular choice being logistic regression. For example, a logistic regression with dichotomous predictors A and B, with interaction term AB and a vector of confounding covariates C.

The model will have the linear equation

$$\mathrm{logit}\left[P\left(Y=1|A=a,\;B=b,\;C=c\right)\right]=\beta_{0}+\beta_{1}a+\beta_{2}b+\beta_{3}ab+\beta_{4}c$$

where Y is the dichotomous outcome and $\exp\left(\beta_{v}\right)$ denotes the odds ratio of each v covariate A,B, and C.

(1) $$\exp\left(\beta_{3}\right)=\mathrm{IMS}=\frac{OR_{A_{1}B_{1}}}{OR_{A_{1}}OR_{B_{1}}}$$

With RERI, the excess risk due to interaction is more than what it would be if the risks were simply additive. This can be assessed on the risk ratio scale using the log-linear model from above:

(2) $$RERI_{RR}=RR_{A_{1}B_{1}}-RR_{A_{1}}-RR_{B_{1}}+1$$

However, if the outcome in question is adequately rare, risk ratios will approximate odds ratios, allowing an assessment on that scale also.

(3) $$RERI_{OR}=OR_{A_{1}B_{1}}-OR_{A_{1}}-OR_{B_{1}}+1$$

In this case, as it is an additive scale, if the OR or RR of the interaction is larger than the combined OR/RR of the standalone variables, a super-additive (RERI > 0) or sub-additive (RERI < 0) interaction will be observed. 

Fitting this in Stata is simple and example code is freely available online, including standard errors calculated using the delta method [34].

#### 4.2.2. Inverse Probability of Treatment Weighting (IPTW)

IPTW can be used when a causal contrast needs to be constructed to create marginal structural models (MSM) [36]. As the basis of our interaction analysis uses a generalized linear model, inverse weighting based on predicting probabilities of counterfactual contrasts can be created. Inverse weighting is a commonly used strategy, our method is described below and an example code for Stata supplied in Text S1.

A treatment of choice was selected first. In this case, the main investigatory factors were race and low SES. These were used analogously to modifiable treatments as they are resultant of environment. As there is a philosophical and ethical problem in predicting biological factors such as race, race was excluded from the prediction of probability.

Stabilized weights were then created by creating probabilities predicted for poverty status. In this study, logistic regression was used as poverty status was dichotomous, however, previous literature notes that even misspecification of models will yield consistent and valid results [37]. The weights are calculated as followed:

(4) $$SW=\frac{P\left(A=a|B=b\right)}{P\left(A=a|B=b,\;C=c\right)}$$

where A is poverty status, B is race and, C are all other confounders. The numerator and denominator can be calculated using simple logistic regressions in most statistical software. The inverse predictions are then applied to all observations where the outcome of poverty is no (*A* = 0), creating the counterfactual contrasts. Weights are then combined with survey weights as a product to create the final stabilized survey weights that can be used to create the marginal structural model [38]. The advantage of marginal structural models is that confounding covariates are no longer a factor as in the creation of the counterfactuals the confounders will be canceled out. 

The marginal structural model was run by a simple regression of the outcome and covariates of interest with the combined weighting applied. As MSM in an interaction analysis was used, the same steps were followed as above to find RERI and multiplicative interaction. If dealing with intervenable factors, a causal inference could be made following this. In this case, as environmental factors were being considered, it would be incorrect to do so. Rather investigation was done using Blinder–Oaxaca decomposition methods to find the intervenable factors that make up the environmental factors. 

#### 4.2.3. Blinder–Oaxaca Decomposition

Blinder–Oaxaca decomposition is a technique that has had extensive use in economics and social science to decompose the differences between two groups of interest. It has a rich history of use in cases of discrimination, with extensive use in investigating the differences in wage gaps between genders, race, and other environmental factors [39]. Historically it has been limited to use in linear regression, however recent development has extended its use to non-linear cases [23,40,41]. This has led to the use of Oaxaca decomposition in areas of epidemiology and health science or other fields with interests in differences between groups. Some examples include decomposing racial differences on anti-obesity medication [42], decomposing socioeconomic status and malnutrition [39], and decomposing change in children’s cognitive function over time [43]. In this study, the technique was applied to investigate the intervenable factors that differentiate Blacks and non-Blacks in terms of oral cancer screening.

At its core, the linear decomposition is constructed by the following equation given an outcome Y predicted by a linear regression of two separate groups A and B, where X is a matrix of variables and β is a matrix of coefficients.

$$Y_{A}=X_{A}\beta_{A}$$

$$Y_{B}=X_{B}\beta_{B}$$

The decomposition is constructed as below [23]:

(5) $$\bar{Y_{A}}-\bar{Y_{B}}=\underset{E}{\underbrace{\left[\bar{F\left(X_{A}\beta_{A}\right)}-\bar{F\left(X_{B}\beta_{A}\right)}\right]}}+\underset{C}{\underbrace{\left[\bar{F\left(X_{B}\beta_{A}\right)}-\bar{F\left(X_{B}\beta_{B}\right)}\right]}}$$

where E is the difference due to characteristics and C is the difference due to coefficients. Within both E and C counterfactual comparisons can be observed. Where for E the characteristics of group B, $X_{B}$ are applied to the coefficients of group A, and for C, the coefficients of group A, $\beta_{A}$ are applied to the characteristics of group B. The caveat is that in a linear model $\bar{Y}=\bar{F\left(X\beta \right)}$ will hold true but this is not the case with non-linear models. In other models, a different mapping of $\bar{Y}$ is required. For creating counterfactual comparisons in logistic models:

$$\bar{Y}=\frac{e^{X\beta }}{1+e^{X\beta }}$$

The choice of reference group will also impact the decomposition. In the above linear example, the reference group is B, with the characteristics comparing the difference as if group A had the same distribution as B, and the coefficients assessing the difference as if B had the same coefficients of A. 

This analysis uses the user-written command mvdcmp to decompose the difference in race on oral cancer screening [23]. The advantage of mvdcmp compared to other commands such as nldecompose or Oaxaca is that it shows decomposition by each variable in both characteristics and coefficients as well as calculating the percentage contribution to the difference. Some disadvantages are that it does not calculate the component of interaction that other methods use [40,41], and decomposition is particularly sensitive to the reversing of reference groups. Nonetheless, the command is both simple to administer and easy to analyze, and its use cases are limitless as long as sufficient difference between groups is found. 

## 5. Conclusions

Our findings show that when removing all measured confounding, being Black and in low SES groups constitutes a sub-additive interaction that decreases the likelihood of receiving oral cancer screening than their individual components combined. In the decomposition between Blacks and non-Blacks in oral cancer screening, higher education and poverty status contribute the most to the explained differences between the two groups. This study has examined how health researchers, epidemiologists, and policymakers can better investigate societal differences in cancer research by using interaction analysis and Blinder–Oaxaca decomposition. As we found in this study the problem of racial differences in health outcomes is not a simple problem to navigate, significant complex mechanisms underly these discrepancies, thus we hope that the methods described above can be used as a tool to both explain, and help bridge the gap as highlighted by the Black Lives Matter movement.

## Figures and Tables

**Table 1:** Descriptive statistics of cohort by non-Black and Black.

Variables	Total *N* = 22,617 (100%)	Non-Black, *N* = 17,489 (77.33%)	Black, *N* = 5128 (22.67%)
General Factors			
Mean Age (SD)	49.77 (17.78)	49.86 (17.95)	49.45 (17.18)
Gender			
Male	48.40	48.54	47.91
Female	51.60	51.46	52.09
SES Factors			
Mean PIR (SD)	2.47 (1.63)	2.53 (1.64)	2.25 (1.58)
Household income			
<$25 k	29.18	27.79	34.04
$25 k–$75 k	42.03	41.64	43.39
$75 k+	28.79	30.57	22.57
Insured			
Yes	80.72	80.41	81.80
No	19.28	19.59	18.20
Education			
Less than HS	22.40	23.30	19.31
HS/GED	52.82	50.08	62.16
College/AA	24.78	26.62	18.53
Health Factors			
Oral cancer screens explained *	
Yes	22.59	21.85	25.42
No	77.41	78.15	74.58
Requires dental work but cannot access	
Yes	22.01	20.86	25.91
No	77.99	79.14	74.09
Oral Cancer Screen			
Yes	18.86	20.46	13.35
No	81.14	79.54	86.65
Currently smokes			
Yes	80.63	18.08	23.75
No	19.37	81.92	76.25

Column percentages listed among characteristics. Survey weighted chi-square tests for categorical variables and a t-test for continuous variables found a meaningful difference between Blacks and non-Blacks, with non-Blacks as a reference in all variables apart from oral cancer screens explained. IR—poverty income ratio, HS—High School, GED—General Educational Development (High School equivalent), AA—Associates Degree.

**Table 2:** Survey weighted multivariate logistic regression with outcome of receiving an oral cancer check, *N* = 9739.

Variables	Odds Ratio (95% CI)	*p*-value
*General Factors*		
Centered Age	1.02 (1.01–1.02)	<0.001
Race		
Non-Black	Reference	
Black	0.40 (0.33–0.49)	<0.001
Poverty		
No	Reference	
Yes	0.71 (0.51–0.98)	0.04
Gender		
Male	Reference	
Female	1.40 (1.21–1.61)	<0.001
*Health Factors*		
Oral cancer checks explained		
No	Reference	
Yes	5.32 (4.47–6.32)	<0.001
Current Smoker		
No	Reference	
Yes	0.91 (0.71–1.18)	0.481
Requires dental work but cannot access		
No	Reference	
Yes	0.73 (0.53–1.00)	0.047
*SES Factors*		
Education		
Less than HS	Reference	
High School/GED	2.43 (1.82–3.24)	<0.001
College/AA	3.61 (2.66–4.90)	<0.001
Household Income		
<25 k	Reference	
25 k–75 k	1.64 (1.23–2.19)	0.001
>75 k	2.41 (1.72–3.38)	<0.001
Insured		
No	Reference	
Yes	1.78 (1.15–2.74)	0.01

Logistic regression was weighted using the appended Mobile Exam Center (MEC) survey weights over 4 National Health and Nutrition Examination Survey (NHANES) cycles.

**Table 3:** Confounded interaction analysis between poverty status and race on the risk of having an oral cancer check, *N* = 9739.

	Non-Black		Black		OR (95% CI) for Race Within Strata of PIR
	*N* Oral Cancer/No Oral Cancer	OR (95% CI)	*N* Oral Cancer/No Oral Cancer	OR (95% CI)	
Not in poverty	2750/9200	1.0	511/2796	0.39 (0.32–0.48)	0.39 (0.32–0.48)
In poverty	220/2347	0.62 (0.44–0.86)	54/870	1.37 (0.75–2.49)	2.21 (1.70–2.89)
OR (95% CI) for PIR within strata of race		0.62 (0.44–0.86)		3.51(2.34–5.19)	

The measure of interaction on additive scale: Relative excess risk due to interaction (RERI) (OR) (95% CI) = 0.32 (0.061–0.58); *p* = 0.016, RERI (RR) not reported due to non-convergence. Measure of interaction on multiplicative scale: ratio of ORs (95% CI) = 1.37 (0.75–2.49); *p* = 0.291. ORs are adjusted for centered age, gender, benefits of cancer checks explained, current smokers, requiring dental work, education level, household income, and insurance status.

**Table 4:** Interaction between poverty status and race on the risk of having an oral cancer check, *N* = 8729.

	Non-Black		Black		OR (95% CI) for Race Within Strata of PIR
	*N* Oral Cancer/No Oral Cancer	OR (95% CI)	*N* Oral Cancer/No Oral Cancer	OR (95% CI)	
Not in poverty	2750/9200	1.0	511/2796	0.37 (0.31–0.43)	0.37 (0.31–0.43)
In poverty	220/2347	1.22 (0.21–6.93)	54/870	0.097 (0.015–0.62)	0.08 (0.012–0.089)
OR (95% CI) for PIR within strata of race		1.22 (0.21–6.93)		0.26 (0.048–1.44)	

Measure of effect modification on additive scale: RERI(OR) (95% CI) = −0.55 (−0.67–−0.42); *p* < 0.001, RERI(RR) (95%CI) = −0.55 (−0.67–−0.42); *p* < 0.001. Measure of effect modification on multiplicative scale: ratio of ORs (95% CI) = 0.097 (0.015–0.62); *p* < 0.001, ratio of RRs (95% CI) = -2.02 (−2.93–−1.11); *p* < 0.001. ORs are inverse probability treatment weighted on poverty status, adjusted for centered age, gender, benefits of cancer checks explained, current smokers, requiring dental work, education level, household income, and insurance status.

**Table 5:** Non-linear Blinder–Oaxaca decomposition of Black-non-Black gap in oral cancer checks, weighted on 8-year Mobile Exam Center (MEC) survey weights, *N* = 23,817.

Difference	Estimate (95% CI)		Percent Contribution	
Raw Difference	−0.26 (−0.29–−0.23)		100	
Explained: Due to difference in characteristics	−0.083 (−0.098–−0.068)		31.99	
Unexplained: Due to differences in coefficients	−0.18 (−0.21–−0.14)		68.01	
	Due to difference in Characteristics		Due to difference in Coefficients	
Variables	Estimate (95% CI)	%	Estimate (95% CI)	%
*General Factors*				
Centered age	−0.016 (−0.034–−0.0018)	6.21	0.00097 (0.00011–0.0018)	−0.37
Gender				
Female	0.00007 (−0.00008–0.0002)	−0.027	−0.025 (−0.063–0.014)	9.43
*SES Factors*				
Education level				
Less than HS	Reference		Reference	
HS/GED	018 (0.0025–0.033)	−6.88	−0.025 (−0.086–0.037)	9.53
College/AA	−0.11 (−0.17–−0.057)	42.91	0.01 (−0.045–0.065)	−3.87
Poverty status				
Yes	−0.033 (−0.055–−0.0099)	12.50	−0.0077 (−0.020–0.0044)	2.96
Household income				
<25 k	Reference		Reference	
25k−75 k	−0.0097 (−0.018–−0.0012)	3.73	−0.087 (−0.13–−0.048)	33.39
75 k+	−0.0048 (−0.055–0.046)	1.84	−0.10 (−0.16–−0.039)	38.60
Insured				
Yes	0.0026 (−0.031–0.036)	−0.99	−0.13 (−0.27–−0.0022)	51.26
*Health Factors*				
Oral cancer checks explained				
Yes	−0.017 (−0.022–−0.011)	6.36	−0.0041 (−0.022–0.014)	1.59
Current Smoker				
Yes	−0.0064 (−0.013–0.0001)	2.46	−0.011 (−0.026–0.0032)	4.29
Requires dental work but cannot access				
Yes	−0.013 (−0.027–0.0015)	4.81	−0.0009 (−0.012–0.0099	0.36
Constant			0.21 (−0.006–0.42)	−79.25

## Data Availability

Publicly available datasets were analyzed in this study. This data can be found here: (https://wwwn.cdc.gov/nchs/nhanes/Default.aspx (accessed on 24 February 2021)).

## Supplementary Materials

The following are available online at https://www.mdpi.com/2072-6694/13/4/848/s1, Text S1: Example of code for creation of Inverse Probability Treatment Weights (IPTW) in Stata.
