# Supplementary material for: Black Lives Matter: A Decomposition of Racial Inequalities in Oral Cancer Screening
Source: Cancers (Basel). 2021 Feb 17;13(4):848. doi: 10.3390/cancers13040848 (PMC7922532; doi:10.3390/cancers13040848)
Supplement: Supplementary file 1 [file cancers-13-00848-s001.pdf]

Supplementary Material

Example of code for creation of Inverse Probability Treatment Weights (IPTW) in Stata

For the construction of stabilized weights as shown in section 4.2.2, we show the formula:

$$SW = \frac{P(A = a|B = b)}{P(A = a|B = b, C = c)} \tag{4}$$

Where A is poverty status (outcome), B is race (treatment/intervention), and C are all other confounders.

The code to create the stabilised weights are as follows:

```
*Creating Inverse Probability Treatment Weights (IPTW)
*Example of creating SW from equation (4)
*Where A = outcome, B = treatment, c = all other confounders

*creating numerator
logit A B
predict x1num
replace x1num = 1-x1num if A==0

*creating denominator
logit A B C
predict x1denom
replace x1denom=1-x1denom if A==0

gen wtx1 = x1num/x1denom

*if using survey weights then multiply wtx1 with survey weights
```
